# A Low Albedo, Thin, Resistant Unit in Oxia Planum, Mars: Evidence for an Airfall Deposit and Late‐Stage Groundwater Activity at the ExoMars Rover Landing Site

**DOI:** 10.1029/2024JE008527

**Published:** 2024-11-22

**Authors:** E. Harris, J. M. Davis, P. M. Grindrod, P. Fawdon, A. L. Roberts

**Affiliations:** ^1^ Department of Science Natural History Museum London UK; ^2^ Department of Earth Science and Engineering Imperial College London London UK; ^3^ School of Physical Sciences The Open University Milton Keynes UK

## Abstract

Oxia Planum, Mars, is the future landing site of the ExoMars *Rosalind Franklin* rover mission, which will search for preserved biosignatures in a phyllosilicate‐bearing unit. Overlying the mission‐important phyllosilicate‐bearing rocks is a dark, capping unit—known here as the Low albedo, Thin, Resistant (LTR) unit—which may have protected the phyllosilicate‐bearing unit over geologic time from solar insolation and radiation. However, little is known about the origin of the LTR unit. Here, we map the LTR unit and investigate its distribution and morphology across 50,000 km^2^ using a variety of orbital remote sensing data sets. The characteristics of the LTR unit include draping palaeo‐topographic surfaces, deposition over a wide elevation range, and a consistent vertical thickness that can be best explained by airfall deposition including a primary or reworked volcanic palaeo‐ashfall. Previous research suggests that the LTR unit was not significantly buried, and we find it to be preferentially preserved with a high mechanical strength in discrete deposits representing palaeo‐topographic lows. We suggest this could be attributed to localized cementation via upwelling groundwater. This scenario suggests that most of the phyllosilicate‐bearing exposures may not have been protected over geologic time, as the uncemented LTR sediment would have easily been removed by erosion. However, our observations indicate that the scarped margins of the LTR unit deposits probably exposed regions of the once protected phyllosilicate‐bearing unit. These areas could be key science targets for the ExoMars *Rosalind Franklin* rover mission.

## Key Points

- We map the Low Albedo, Thin, Resistant (LTR) unit, finding stratigraphic relationships to the mission‐important phyllosilicate‐bearing unit
- We determine that the LTR unit is an airfall deposit, with a possible volcanic source, that fell between the late‐Noachian and early‐Hesperian
- We found that preservation of the LTR unit was driven by groundwater cementation in an otherwise arid environment

## 1 Introduction

Oxia Planum (18.20°N, 335.45°E) has been chosen as the landing site of the European Space Agency (ESA) ExoMars *Rosalind Franklin* rover with the mission goal of searching for signs of past or present life (Quantin‐Nataf et al., 2021; Vago et al., 2017). Oxia Planum contains evidence for ancient aqueous activity, including a sediment fan in the east of the landing ellipse that may record numerous episodes of fluvial activity (Fawdon et al., 2022). Approximately 35% of the landing ellipse contains exposed phyllosilicate‐bearing rocks (Mandon et al., 2021; Parkes‐Bowen et al., 2022) detected from orbit by the Compact Reconnaissance Imaging Spectrometer for Mars (CRISM; Murchie et al., 2007) and Observatoire pour la Minéralogie, l'Eau, les Glaces et l'Activité (OMEGA; Bibring et al., 2005) instruments. The phyllosilicate‐bearing unit is the main target for the mission as it has the potential to contain preserved biosignatures (Brossier et al., 2023; Quantin‐Nataf et al., 2021; Summons et al., 2011). The rover will address this objective by drilling up to 2 m into the sub‐surface to provide a core sample for analysis (Vago et al., 2017).

The key to long‐term protection of possible remnant organics within the phyllosilicate‐bearing unit at Oxia Planum is the presence of a low albedo geologic unit, relative to the underlying, lighter‐toned phyllosilicate‐bearing unit, forming a local cap in many areas. This capping unit has previously been described as an “Amazonian dark resistant unit; ADRU” (Mandon et al., 2021; Quantin‐Nataf et al., 2021), “dark capping terrain” (Fawdon et al., 2021), “dark capping unit” (McNeil et al., 2021), and most recently as the “overlying Dark material; oDm” (Fawdon et al., 2024). Here, we refer to this as the Low albedo, Thin, Resistant (LTR) unit in order to separate our observations and interpretations from previous conclusions. This is the first study to focus solely on the analysis of the LTR unit. We used orbital remote sensing data to assess the morphology, distribution, and stratigraphy of the LTR unit at Oxia Planum. Previous work (e.g., Quantin‐Nataf et al., 2021) has highlighted the importance of the resistant, capping qualities of the LTR unit; however, despite its significance, its origin is largely unconstrained. It has been suggested that the LTR unit may have formerly been more extensive (Quantin‐Nataf et al., 2021) and has been previously interpreted as a lava flow (Gary‐Bicas & Rogers, 2021; Ivanov et al., 2020; Mastropietro et al., 2020; Pajola et al., 2017; Quantin‐Nataf et al., 2021) as well as sedimentary deposits possibly from a palaeo‐lake or playa environment (Fawdon et al., 2022). Our assessment includes comparisons to possible analogous geologic units on both Earth and Mars. We then assess the potential methods of LTR unit lithification and the implications this has for the palaeo‐environment of Oxia Planum.

## 2 The Regional Geology of Oxia Planum

**Figure 1:**
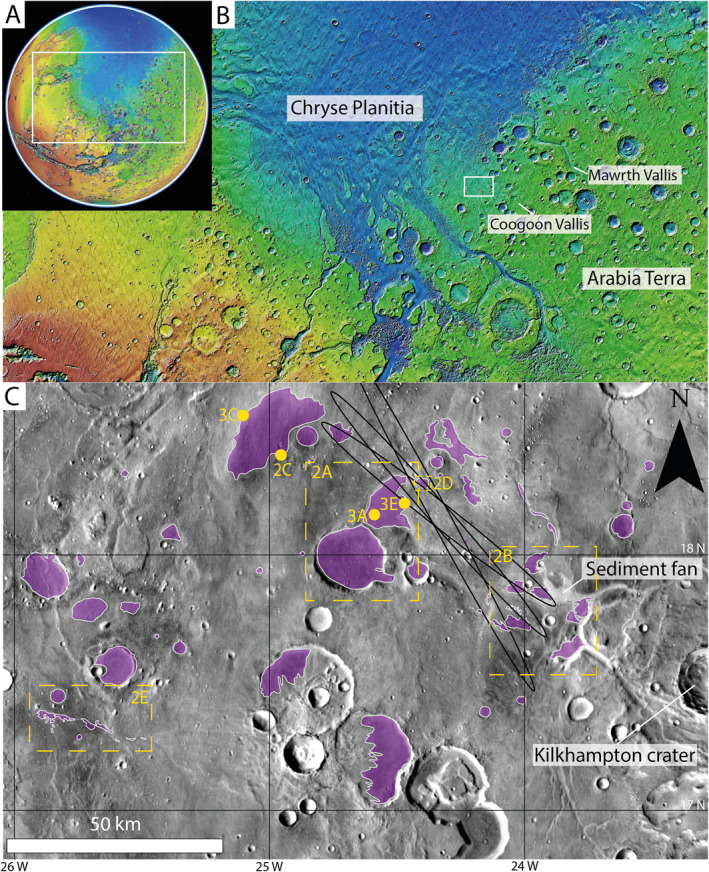
Our study area centered around the landing site of the ExoMars *Rosalind Franklin* rover at Oxia Planum (18.3°N, 335.4°E). (a) Mars Orbiter Laser Altimeter (MOLA; Zuber et al., 1992) global map highlighting western Arabia Terra and Chryse Planitia. (b) Regional MOLA map of western Arabia Terra showing the study area of Oxia Planum, as well as regional features of interest including Coogoon and Mawrth Valles. (c) The LTR unit (purple) mapped at 1:50,000 using Context Camera (CTX; Malin et al., 2007) data displayed on a Thermal Emission Imaging System (THEMIS; Christensen et al., 2004) daytime background. Key features of interest highlighted include the ExoMars landing ellipses where multiple ellipses represent the areas of landing throughout the 2022 launch window, Kilkhampton crater, the sediment fan, and a possible volcaniclastic source. Locations of future figures are identified in yellow.

Our study region is centered on Oxia Planum situated on the border between the cratered, Noachian‐age highlands of north‐western Arabia Terra, and the Hesperian‐age plains of Chryse Planitia in the northern lowlands (Figure 1) (Tanaka et al., 2014). We have mapped the LTR unit over a 50,000 km^2^ region of Oxia Planum, but it is noted here that units exhibiting similar morphologies as the LTR unit do exist beyond the scope of our study region in the wider Arabia Terra region (Noe Dobrea et al., 2010). Arabia Terra is the northern most region of the cratered highlands and contains distinctive, layered sedimentary units (e.g., Annex & Lewis, 2020; Lewis & Aharonson, 2014; Pondrelli et al., 2015; Schmidt et al., 2021; Zabrusky et al., 2012), evidence of widespread ancient fluvial systems (Davis et al., 2016, 2019; Fawdon et al., 2022; Molina et al., 2017), as well as unusual depressions interpreted as ancient explosive volcanoes that are a potential volcaniclastic source for the extensive sedimentary units (Brož et al., 2021; Chu et al., 2023; Michalski & Bleacher, 2013; Whelley et al., 2022).

There is an abundance of evidence for regional palaeo‐fluvial activity including Coogoon and Mawrth Valles (Figure 1). Mawrth Vallis contains a large phyllosilicate‐bearing unit (Loizeau et al., 2007; Lowe et al., 2019; Wray et al., 2008) and dark deposits within palaeo‐topographic lows (Noe Dobrea et al., 2010)—a stratigraphy similar to Oxia Planum—and was previously shortlisted as a candidate landing site for the ExoMars *Rosalind Franklin* rover (Poulet et al., 2020). Coogoon Vallis is a large (10 km wide) channel system that terminates with a sediment fan in east Oxia Planum (Figure 1). The sediment fan itself has been previously dated at >3.7–3.4 Ga based on the use of crater size‐frequency distribution (CSFD) to date the ejecta of the superposed Kilkhampton crater (Gary‐Bicas & Rogers, 2021) and has been interpreted as a delta (Fawdon et al., 2022; Quantin‐Nataf et al., 2021). This system records fluvial processes, erosion, and transport from Arabia Terra to Oxia Planum (Molina et al., 2017). Stratigraphic interpretations of the channels in the system suggest that Oxia Planum experienced multiple aqueous episodes including the development of Coogoon Vallis in the Noachian‐Hesperian and the latter incision of channels (Fawdon et al., 2022). In addition, a network of inverted channels (Fawdon et al., 2022) interbedded within the phyllosilicate‐bearing unit (Davis et al., 2023) are evidence of ancient, widespread fluvial activity and extensive erosion at Oxia Planum exposing these features in inverted relief. Evidence for large‐scale erosion can also be interpreted from the distribution and morphology of a large number of mounds up to 500 m high. These mounds likely preserve the former vertical extent of eroded units that extended around the rim of Chryse Planitia prior to mass erosion (McNeil et al., 2021).

In this section, we summarize the geology of Oxia Planum that has been previously extensively investigated (e.g., Fawdon et al., 2024; Quantin‐Nataf et al., 2021). The oldest exposed rocks comprise a ∼4 Ga phyllosilicate‐bearing unit, as determined using CSFD in previous research (Quantin‐Nataf et al., 2021), overlain by various light‐ and dark‐toned deposits including a dark, mantling deposit identified with a maximum thickness of ∼5 m that drapes over the phyllosilicate‐bearing unit (Quantin‐Nataf et al., 2021). The LTR unit sits largely at the top of the stratigraphy at Oxia Planum, post‐dating the depositional and erosional events that created the mounds (McNeil et al., 2021). Previously, the LTR unit has been dated at 2.6 Ga using CSFD, but the authors note the ambiguity of the small surface area of the deposits and postulate that a higher age is not out of the question (Quantin‐Nataf et al., 2021). The LTR unit can be differentiated from the dark, mantling deposit as it appears to have a larger vertical extent of ∼20 m and is more resistant to erosion with a rugged, pitted surface and a high abundance of boulders (Pajola et al., 2017).

The orbital identification of approximately 2,500 km^2^ of phyllosilicate‐bearing unit was key to the selection of Oxia Planum as the landing site of the ExoMars *Rosalind Franklin* rover (Mandon et al., 2021) due to the possibility of enhanced organic preservation here (Vago et al., 2017). Orbital studies have shown the phyllosilicate‐bearing unit to be at least 50–100 m thick (Parkes‐Bowen et al., 2022). Hyperspectral imaging from the CRISM instrument has identified Fe/Mg‐ phyllosilicates, showing close spectral matches to vermiculite and smectite (Brossier et al., 2022; Mandon et al., 2021; Quantin‐Nataf et al., 2021). It has yet to be established if the phyllosilicate‐bearing unit is detrital or authigenic, but favored formation theories include subaqueous deposition—for example, palustrine, lacustrine, or marine environments—or subaerial sediments altered by groundwater (Mandon et al., 2021; Quantin‐Nataf et al., 2021). Analysis of high‐resolution data including the High Resolution Imaging Science Experiment (HiRISE; McEwen et al., 2007) color images and the multi‐spectra capabilities of the Color and Stereo Surface Imaging System (CaSSIS; Thomas et al., 2017), has identified the phyllosilicate‐bearing unit stratigraphy to comprise relatively orange‐toned and lighter‐toned sub‐units (Fawdon et al., 2024). The apparent compositional variation in the sub‐units may arise due to varying mineralogy, exposure, and/or dust coverage (Mandon et al., 2021; Parkes‐Bowen et al., 2022). The LTR unit sits directly above the phyllosilicate‐bearing unit within the stratigraphy and may have once covered a larger extent of Oxia Planum than we see today (Quantin‐Nataf et al., 2021), acting as a cap to protect the phyllosilicate‐bearing unit from solar insolation and radiation, potentially increasing the chances of organic matter preservation and detection by the ExoMars *Rosalind Franklin* rover (Quantin‐Nataf et al., 2021).

Hyperspectral imagery from the CRISM and OMEGA instruments has identified mafic spectral signatures associated with the LTR unit at a scale of 18 m/pixel (Mandon et al., 2021; Quantin‐Nataf et al., 2021). Whilst first‐order interpretations may suggest a volcanic origin (Mandon et al., 2021), a mafic spectral signature is typical of dark toned Mars sands due to the erosion of basalt, which high resolution studies observe to make up many of the dark toned deposits in Oxia Planum (Fawdon et al., 2021, 2024). Consequently, this observation may not be informative regarding the origin of the LTR unit. Interestingly, phyllosilicate spectral signatures have also been identified in CRISM data associated with the LTR unit (Gary‐Bicas & Rogers, 2021), and ultimately the question of the origin of the LTR unit remains unanswered.

## 3 Data and Methods

We investigated the LTR unit at Oxia Planum using a variety of orbital remote sensing data sets. The mapping of the LTR unit was undertaken using Context Camera (CTX; Malin et al., 2007) images at 5–6 m/pixel. This included the use of a CTX orthorectified image (ORI) mosaic from Fawdon et al. (2021). To investigate the detailed morphology of the LTR unit at higher resolution, HiRISE images (McEwen et al., 2007) (25–50 cm/pixel) were analyzed. Exploration of color and tonal variations within the LTR deposits was assessed in CaSSIS (Thomas et al., 2017) Near‐Pan Blue (NPB) and Red Green Blue (RGB) composite images, including a CaSSIS RGB mosaic from Fawdon et al. (2021). To explore 3D views and analyze topographic profiles, HiRISE and CTX Digital Terrain Models (DTMs) were utilized, which were created in previous research (Fawdon et al., 2021; Tao et al., 2021) with spatial resolutions of 12 m (CTX) and 50 cm (HiRISE). All image IDs used in the mapping and exploration of the LTR unit at Oxia Planum can be found embedded within figure captions throughout. Measurements of topography were supplemented where needed by the Mars Orbiter Laser Altimeter (MOLA; Zuber et al., 1992) and High Resolution Stereo Camera (HRSC; Neukum et al., 2004) data (Tao et al., 2021), the results of which can be found in Table S1.

**Figure 2:**
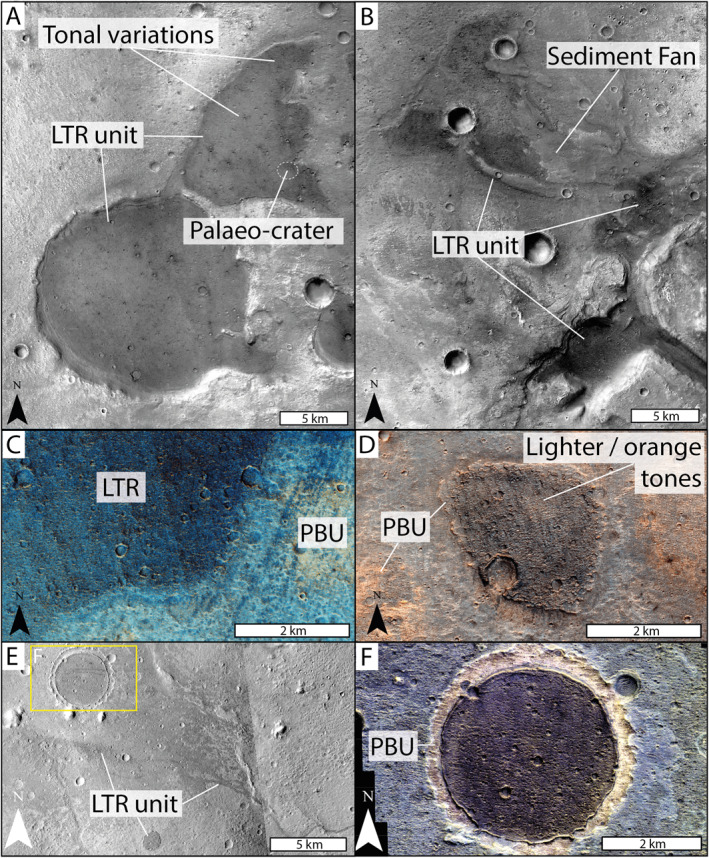
The LTR unit observed within a variety of remote sensing data sets. CTX and CaSSIS ORI mosaics are from Fawdon et al. (2021). (a) The two largest LTR deposits in the study area (CTX ORI mosaic). (b) LTR deposits in contact with sediment fan deposits (CTX ORI mosaic). (c) The LTR unit and phyllosilicate‐bearing unit (PBU) seen in CaSSIS NPB ORI. (d) A smaller LTR outcrop ∼2 km in diameter as seen in CaSSIS RGB ORI. Here we note the identification of relatively orangey‐hues, similar to the surrounding phyllosilicate‐bearing unit (PBU). (e) The LTR unit preserved in an inverted crater (see “F”) and overlying inverted sinuous ridges (CTX: D02_028130_1972, G23_027009_1981). (f) The LTR unit forming an inverted palaeo‐crater surrounded by an inlying “moat” of phyllosilicate‐bearing unit (CaSSIS NPB; MY36_016394_162).

**Figure 3:**
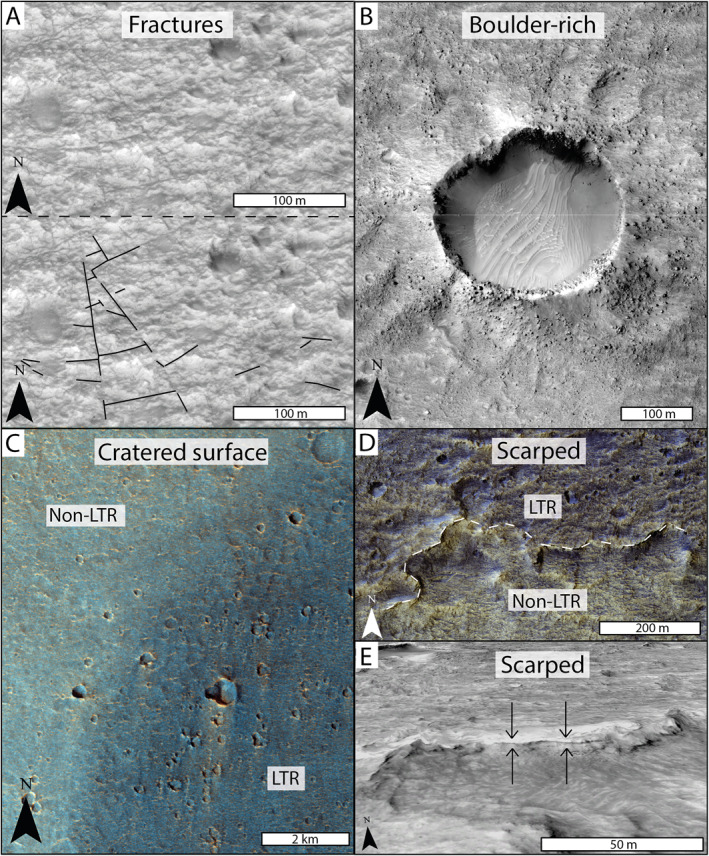
Morphological attributes of the LTR unit. (a) Polygonal fractures identified within the LTR unit (HiRISE: ESP_0448111_1985). (b) A ∼200 m diameter impact crater formed into the LTR unit surrounded by boulder‐rich ejecta. (c) The LTR unit preserves a higher density of craters in comparison to non‐LTR unit surfaces (CaSSIS NPB ORI; Fawdon et al., 2021). (d) The scarped edge of the LTR unit has been highlighted. Here, we note the apparent diffuse and gradual color change from the phyllosilicate‐bearing unit to the LTR unit (HiRISE: PSP_009735_1985, ESRI color stretch). (e) HiRISE DTM 3D view of the scarped edge of the LTR unit. Possible internal layering has been highlighted with the arrows (HIRISE DTM: Tao et al., 2021; HiRISE image: PSP_009880_1985).

**Figure 4:**
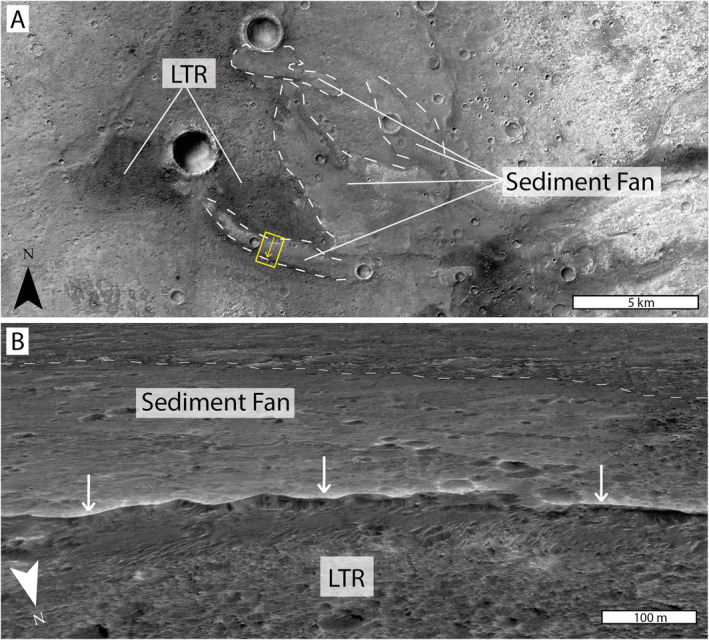
The stratigraphic relationship between the LTR unit and the sediment fan at Oxia Planum. (a) The eroded sediment fan in Oxia Planum (outlined) and LTR unit deposits in contact with the sediment fan. The yellow box shows the location and viewing direction of panel “B” (CTX ORI; Fawdon et al., 2021). (b) Further analysis of the contact between the sediment fan overlying the LTR unit. The low‐albedo, rough surface of the LTR unit can be seen in the foreground prior to the positive relief scarp of the sediment fan deposit occurs. The edge of the sediment fan in the background is highlighted. This 3D view is composed of a HiRISE DTM (Tao et al., 2021) and overlain orthoimage ESP_039932_1980.

**Figure 5:**
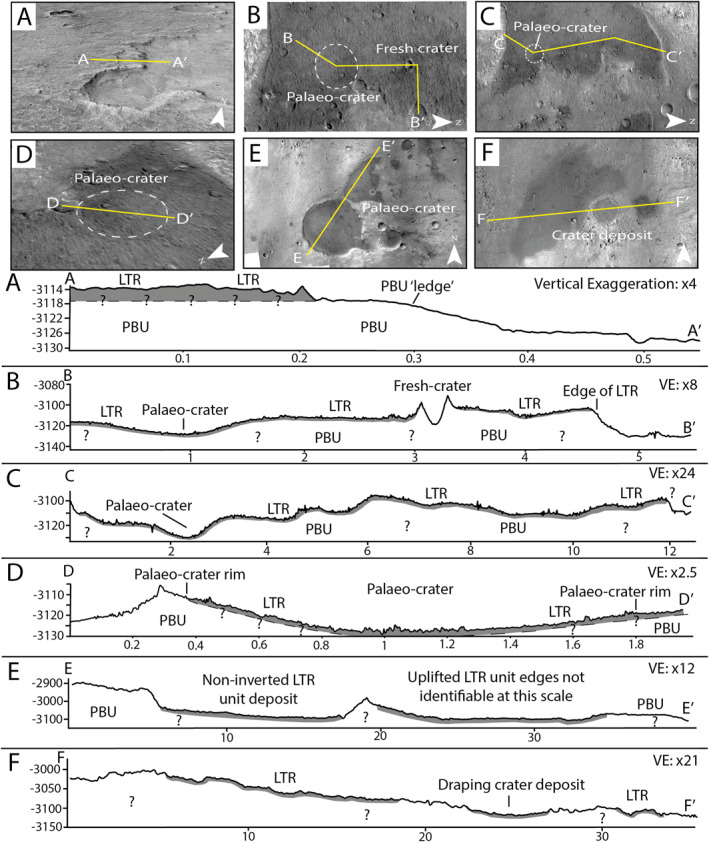
Analysis of the LTR unit within topographic profiles. The *x*‐axes display elevation relative to the Mars datum, and the *y*‐axes show distance in km. Note the varying vertical exaggeration (VE). The LTR unit is in gray, but it is noted that the apparent vertical thickness is based on the analysis of the A‐A′ profile (in‐text) and is not scaled throughout. Profiles (a–d) were created using HiRISE DTM (Tao et al., 2021), and profiles (e and d) were created using CTX ORI DTM (Fawdon et al., 2021). (a) The majority of the positive relief of the LTR unit can be attributed to the underlying phyllosilicate‐bearing unit (PBU). (b) The LTR unit drapes the palaeo‐crater in comparison to the fresh‐crater deposit that does not contain the LTR unit. (c) Topographic variations across one of the largest LTR unit deposits highlight the draping qualities of the LTR unit. (d) Detailed analysis of the palaeo‐crater that has preserved its bowl‐like morphology due to the draping of the LTR unit. (e) Analysis of the two largest LTR unit deposits shows one of the deposits is not inverted, but overall the presence LTR unit does not make a significant difference to the topography of the landscape. (f) A non‐crater LTR deposit showing the thin, draping nature of the LTR unit. The crater in the profile further highlights the draping quality expressed.

All mapping and image analysis was performed within the Geographic Information System (GIS) software ArcGIS Pro 2.8. The LTR unit was mapped at a scale of 1:50,000 using CTX data, and the results displayed on a Thermal Emissions Imaging System (THEMIS; Christensen et al., 2013) Daytime mosaic basemap (Figure 1c). The morphology, geological characteristics, and tonal variations of the LTR unit were investigated using CTX, HiRISE, and CaSSIS images including various image stretches (e.g., Figures 2 and 3—see figure captions for details). Finally, stratigraphic relationships between geological units at Oxia Planum (Figures 4 and 5) were investigated using topographic profiles and elevation data from HiRISE, CTX, and MOLA DTMs. Further examination of the hyperspectral data sets available to investigate the LTR unit, beyond those previously published for example, (Mandon et al., 2021; Quantin‐Nataf et al., 2021), is beyond the scope of this investigation.

## 4 The Morphology and Distribution of the LTR Unit

In panchromatic CTX and HiRISE images, the LTR unit is observed as discrete, low albedo, irregular outcrops (Figures 2a, 2b, and 2e). The outcrops are sometimes observed to be circular, or near‐circular, suggesting they were originally deposited as the fill of now inverted impact craters (Figure 2f). The LTR unit is also identified to cap sinuous ridges (Figure 2e) interpreted to be inverted fluvial channels (Davis et al., 2023). CTX and HiRISE imagery shows intra‐outcrop tonal variations, with relatively lighter‐tones occasionally observed (Figures 2a and 2d). In CTX imagery, the LTR unit appears fairly monochromatic (Figures 2a, 2b, and 2e) but in CaSSIS NPB data the LTR unit exhibits deep blue‐black tones that vary across the deposit (Figures 2c and 2f). CaSSIS RGB data also show occasional and inconsistent observations of relatively orange tones, similar in color to the surrounding phyllosilicate‐bearing unit (Figure 2d). Analysis of HiRISE imagery reveals polygonal fracturing that can be identified throughout the LTR unit deposits (Figure 3a). The fractures are often associated with locations of lighter‐areas, although this may be an observation bias associated with ease of feature identification within the lighter toned areas. This fracturing might be a surface expression of the underlying phyllosilicate‐bearing unit expressed in areas of thinning LTR unit coverage, as noted in Quantin‐Nataf et al. (2021) and Parkes‐Bowen et al. (2022). However, the fracturing could also be related to diagenetic burial processes post‐dating lithification. The LTR unit has a high mechanical strength as noted by meter‐scale boulders strewn across the surface, with higher concentrations observed around impact craters (Figure 3b). Qualitatively, the LTR unit has a high crater retention rate in comparison to other units in the study area (Figure 3c), further supporting the LTR unit having a high mechanical strength.

Stratigraphically, the LTR unit is found to directly, and uncomfortably, overlie the Noachian‐aged phyllosilicate‐bearing bedrock units (McNeil et al., 2021; Parkes‐Bowen et al., 2022; Quantin‐Nataf et al., 2021). This stratigraphic relationship can be identified within the scarped edges of the LTR unit deposits (Figure 3d), where we see meter‐decameter scale vertical cliff faces exposing what appears to be the contact with the underlying lighter‐toned phyllosilicate‐bearing unit in a largely planar boundary (Figure 3e). Within LTR unit outcrops, we see relatively lighter, orange tonal variations expressed in CaSSIS data similar to the surrounding phyllosilicate‐bearing unit tones (Figure 2d). We have also identified examples of a sediment fan overlying the LTR unit in eastern Oxia Planum (Figure 4). Here, CTX images show the four distinct, light‐toned branches of the sediment fan. The southernmost branch of the sediment fan is shown to overlie the LTR unit. HiRISE DTM analysis shows a definite scarp on the southern side of the branch, where the sediment fan sits on top of the LTR unit (Figure 4b). We were not able to distinguish instances of the LTR unit in contact with any other part of the sediment fan and we do not observe examples of the LTR unit above the sediment fan in any location. Therefore, we postulate that the LTR unit was either deposited prior to the formation of the sediment fan, previously dated at >3.7–3.4 Ga (Gary‐Bicas & Rogers, 2021), or during a hiatus in the activity associated with the sediment fan. Based on the relative stratigraphic relationships to the underlying phyllosilicate‐bearing unit and the overlying sediment fan, we are able to date the LTR unit to between the late‐Noachian and the early‐Hesperian (∼4–3.7 Ga).

We measured the LTR unit thickness using available HiRISE and CTX DTMs where there was appropriate exposure. These data are displayed graphically in Figure 5. The topographic profile A‐A′ is 0.6 km in length and highlights the surface variability from the basement phyllosilicate‐bearing unit across the edge of the LTR unit deposit (Figure 5a). Here, we were able to capture the relative vertical relief associated with both the LTR unit and the underlying phyllosilicate‐bearing unit. A “ledge” of phyllosilicate‐bearing rocks comprises ∼8 m of the vertical relief across ∼0.2 km, and we observe the LTR unit expressing ∼3–4 m of positive relief on top of this. We suggest that previous analysis of LTR unit thickness (e.g., Pajola et al., 2017) likely recorded the vertical extent of the underlying phyllosilicate‐bearing unit in their assessment. Our analysis of the A‐A′ profile assumes a planar, horizontal, and continuous contact between the phyllosilicate‐bearing unit and the LTR unit. Analysis of subsequent topographic profiles in Figure 5 includes the LTR unit thickness measurement of ∼3–4 m obtained in profile A‐A′. Figures 5b–5d show topographic profiles through a ∼1.5 km diameter palaeo‐crater located within the LTR‐unit deposit near the western scarped edge of the largest LTR unit deposit that was likely emplaced prior to the deposition of the LTR unit. The location of this palaeo‐crater can be identified in Figure 2a where the rim of the palaeo‐crater is visible. The B‐B′ profile shows a bowl‐like morphology associated with this palaeo‐crater, and we do not observe an increase in the LTR unit thickness toward the center of the palaeo‐crater suggesting the LTR unit drapes the topography here along the contours of the crater. Profiles B‐B′ and C‐C′ highlight the wider topographic variations and undulations within one of the largest LTR unit deposits here shown in Figures 2b and 2c. The D‐D′ profile shows that there is a ∼10 m increase in relief at the edge of the LTR unit deposit here, possibly owing to the uplift associated with the rim of the palaeo‐crater in combination with the positive relief of the underlying phyllosilicate‐bearing unit. Finally, topographic profiles E‐E′ and F‐F′ show a wider regional scale where the LTR units appear largely flat, often not affecting the wider topography (Figures 5e and 5f). These observations illustrate the consistently thin vertical thickness of the LTR unit. Interestingly, all topographic profiles assessed highlight the draping nature of the LTR unit across the Oxia Planum.

**Figure 6:**
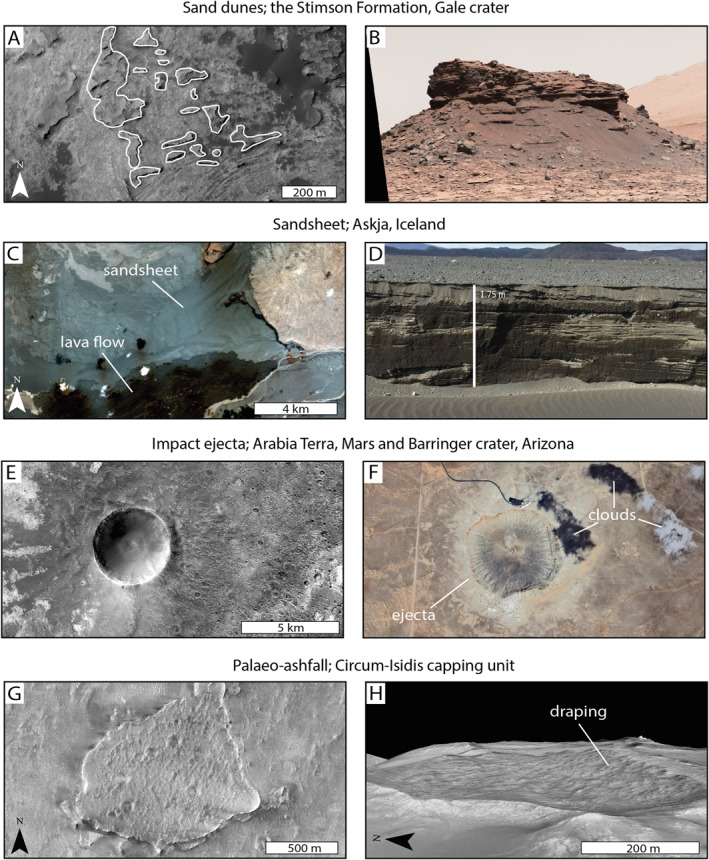
Potential analogs for the LTR unit on Earth and Mars. (a) Murray Buttes in Gale crater capped with the Stimson Formation (HiRISE: ESP_043539_1755). The butte locations have been outlined in white in accordance with Banham et al. (2021) (b) MSL *Curiosity* rover MastCam mosaic of a Murray Butte containing a Stimson formation sandstone cap (PIA20841. Credit: NASA/JPL‐Caltech/MSSS). (c) Orbital view of the Askja sandsheet, Iceland overlain by the 2014–2015 Holuraun eruption (LANDSAT image LC09_L2SP_217014_20220904_20230330_02_T1). (d) The lower section of the Askja sandsheet, Iceland is cemented (Image courtesy of I. Ukstins). (e) Impact ejecta in Arabia Terra (CTX: D16_033404_1999). (f) The extent of the ejecta blanket at Barringer (Meteor) crater (Image: Google Earth). (g) The circum‐Isidis capping unit as seen from orbital data (HiRISE: ESP_016931_1980). (h) 3D view of the circum‐Isidis capping unit draping the topography (HiRISE DTM DTEEC_002888_2025_002176_2025 and orthoimage PSP_002888_2025).

The size of the LTR unit outcrops ranges from 0.5 to 180 km^2^, totaling 450 km^2^ over a study region of 50,000 km^2^ (Figure 1c). At present, the volume of the LTR unit is on the order of 1.35 × 10^6^–1.80 × 10^6^ m^3^, assuming a consistent unit thickness of 3–4 m (Figure 5a), but this may have been at least 2.25 × 10^10^ m^3^ if the LTR unit once continuously blanketed the entire study area as suggested by previous work (Quantin‐Nataf et al., 2021). The LTR unit deposits become smaller and less frequent the further away from the center of the study area searched and are not found to be present east of Kilkhampton crater or into the Chryse Planitia basin (Figure 1). We have decided to distinguish the LTR unit from other dark, capping units of the region based on this spatial distinction, although we note that units similar in morphology to the LTR unit are present at Mawrth Vallis (Noe Dobrea et al., 2010) and within the wider Arabia Terra region (Fassett & Head III, 2007), although these deposits are more spatially inconsistent and can often be associated with large impact ejecta blankets (e.g., Figure 6g). The distribution of the LTR unit outcrops covers an elevation range of 470 m (−3123 to −2653; Table S1). The LTR unit outcrops are largely preferentially preserved in palaeo‐topographic lows including, but not limited to, ancient impact craters, some of which have since been inverted (Figure 2f), and on top of sinuous ridges interpreted as inverted channels (Davis et al., 2023; Fawdon et al., 2022) (Figure 2e), both of which have been subjected to terrain inversion.

## 5 The Possible Formation Mechanisms of the LTR Unit

Our analysis of the LTR unit has shown it to have distinct morphological characteristics within Oxia Planum, including the formation of local caps, a low albedo, a high mechanical strength, consistent but low vertical thickness, preferential preservation in palaeo‐topographic lows, and draping palaeo‐topography. The spatial extent of the LTR unit deposits we see today likely does not resemble the initial depositional extent of this unit, as it may have once extended across the entirety of the study area (Quantin‐Nataf et al., 2021). This section discusses possible LTR unit formation mechanisms by comparing and contrasting the LTR unit with similar geological materials on Earth and Mars (Figure 6). A summary of this discussion can be found in Table 1.

**Table 1:** A Range of Geologic Units for Comparison With the LTR Unit

		Morphological and distributive traits			
Deposit origin	Deposit type	Deposited over wide geographical range (∼50,000 km^2^ +)	Consistent low vertical thickness	Drapes over topography	Deposited over wide elevation range (∼500 m +)
Volcanic	Lava flow				
Volcanic	Pyroclastic flow				
Volcanic	Ashfall	✓	✓	✓	✓
Impact	Distal ejecta	✓		✓	✓
Impact	Impact melt				
Aeolian	Dunes		✓		
Aeolian	Sand sheet	✓	✓		
Aeolian	Airfall	✓	✓	✓	✓
Aqueous	Fluvio‐lacustrine		✓		
Aqueous	Marine				
Aqueous	Tsunami	✓		✓	✓
?	LTR Unit	✓	✓	✓	✓

*Note*. The LTR unit (bottom row) indicates a presence of a wide geographic range, consistent low vertical thickness, topographic draping, and a wide elevation range. A volcanic ashfall and airfall deposits are the only deposits identified here to contain all the morphologic and distributive traits as the LTR unit.

Here, an aeolian origin is considered for the LTR unit. The development of aeolian sand seas, or ergs—wind‐blown sand including dune, fields, and sand sheets—can produce areally extensive deposits reaching more than 1 × 10^5^ km^2^ (e.g., Breed et al., 1987; Purdie, 1984). On Earth and Mars, sandstone deposits preserve both sand dunes and sandsheet environments, as identified within the geologic record (Banham et al., 2018, 2021; Chojnacki et al., 2020; Day et al., 2019; Kocurek & Day, 2018; McKee, 1966; Trewin, 1993). On Mars, aeolian deposits have been identified to exhibit mafic spectral signatures, similar to the LTR unit, as they are primarily comprised of eroded basaltic components (Ehlmann & Edwards, 2014). Palaeo‐dune fields are capable of forming meter‐scale thick capping units, characteristic of the LTR unit, as identified in the Stimson formation in Gale crater (Figures 6a and 6b; Banham et al., 2021). Mars Science Laboratory (MSL) *Curiosity* rover ground‐based images of the Stimson formation show internal cross‐bedding associated with the migration of dunes preserved in the capping units (Figure 6b; Banham et al., 2018). Internal sedimentary structures have not been observed in the LTR unit from orbital data so far, and our current data resolution shows the LTR unit as apparently structureless (Figure 3e). Aeolian analogs on Earth include the Askja sandsheet, Iceland, that exhibits scarped edges and a unit height of ∼10 m (Figures 6c and 6d) (Mountney & Russell, 2004). However, importantly the Askja sandsheet fills topographic lows (Mountney & Russell, 2004), whereas the LTR unit is shown to drape topographic variations (Figure 5). Although terrestrial research has identified some environments in which dunes and sandsheets drape valleys and climb topography (Dong et al., 2018; Hay et al., 2021), these often do so based on wind regimes, leading to asymmetrical deposits that typically accumulate in the downwind face of a depression. In Oxia Planum, the LTR unit drapes topographic variations with high levels of symmetry, for example, appearing to have been deposited evenly on all sides of crater walls (Figure 5d). Due to this, we do not favor the LTR unit to have had an aeolian origin.

Another possible interpretation of LTR is that it is a distal ejecta or melt associated with a large, nearby crater. Large impacts may have been able to produce enough melt to exceed the crater volume (Manske et al., 2021; Osinski, 2004), but impact melts are generally geographically confined to their origin crater (Osinski, 2004). Ejecta blankets, however, have more extensive features. Proximal ejecta are often comprised of larger, blocky fragments and distal ejecta (occurring >2.5 crater diameters away from its source crater) is a thin, discontinuous layer comprised of impact spherules (Glass & Simonson, 2012). The thin, draping morphology of the LTR unit may be similar to traits of distal impact ejecta (Housen et al., 1983; Osinski, 2004); however, the boulder‐rich nature of the LTR unit (Pajola et al., 2017) suggests it may be proximal ejecta. The major uncertainty with this hypothesis is the wide distribution range of the LTR unit and the lack of any obvious source craters. Within western Arabia Terra, there are a number of impact craters more than 100 km in diameter that have the potential to form a global distribution of ejecta (Segura et al., 2002) including Galilaei, Trouvelot, Rutherford, and Becquerel craters. Examples of impact ejecta in Arabia Terra (Figure 6e) show low albedo capping units, similar to the LTR unit. However, these are easily traced back to a source crater, which we cannot do with the LTR unit, suggesting that it is not impact‐related.

Here, we discuss the possibility that the LTR unit formed in an aqueous depositional environment. Previous research has interpreted the LTR unit as having formed through palaeo‐lake or playa‐like processes, based on the association of LTR deposits with palaeo‐craters that contain evidence for lakes as well as inverted fluvial channels (Fawdon et al., 2022). We observe the LTR unit to be deposited discretely over ∼50,000 km^2^ but may have once extended throughout the study area (Quantin‐Nataf et al., 2021). It is possible that there was once a standing body of water present at Oxia Planum ≥3.7 Ga as the sediment fan (dated via Kilkhampton crater ejecta CSFD dating, Gary‐Bicas & Rogers, 2021) has been previously interpreted as a remnant delta deposit (Fawdon et al., 2022; Quantin‐Nataf et al., 2021). Our investigation of the stratigraphy of Oxia Planum shows that the LTR unit is below at least one branch of the sediment fan (Figure 4), suggesting that its deposition occurred prior to the last aqueous episode. Further evidence of this is provided by the lack of hydrous alteration identified within the spectral signatures of the LTR unit in previous research (Gary‐Bicas & Rogers, 2021; Quantin‐Nataf et al., 2021). If a large, regional lake was present at Oxia Planum, we might expect larger scale preservation of the LTR unit, as cementation would have occurred throughout, or we might observe structures associated with lacustrine deposition, including the filling of topographic lows. Furthermore, we did not observe any evidence of an interbedded stratigraphic relationship with the sediment fan, as might be expected if the LTR unit were associated with lacustrine deposition. Instead, we observe strict boundaries between the two units suggesting two separate depositional events and environments.

We also postulate the possibility that the LTR unit could be a deep‐sea or tsunami deposit. A deep marine environment at Oxia Planum is unlikely as suggested palaeo‐shorelines within the northern lowlands are all higher in latitude and lower in elevation than Oxia Planum (Carr & Head III, 2003; Ivanov et al., 2017; Sholes et al., 2021). However, the proximity of Oxia Planum to putative shorelines means a tsunami induced deposition is not out of the question. A candidate tsunami‐forming event is the Lomonsov impact crater that formed in the late‐Hesperian to early‐Amazonian. The event is suggested to have occurred in an aqueous environment and may have resurfaced the northwestern Arabia Terra with tsunami deposits (Costard et al., 2019). The boulder‐rich nature of the LTR unit (Figure 3b) could represent large, detrital boulders transported and deposited within a high‐energy event. However, we see an increase in boulder‐rich areas around impact craters within the LTR unit, suggesting that the boulders are associated with the mechanical strength of the LTR unit and produced by impacts, as opposed to eroded out detrital sediments. Thus, the LTR unit is unlikely to be of aqueous origin.

The LTR unit has previously been interpreted as a lava flow due to its low albedo, massive appearance, presence of apparent lobate scarps, and the orbital detection of mafic spectral signatures (Gary‐Bicas & Rogers, 2021; Ivanov et al., 2020; Mastropietro et al., 2020; Pajola et al., 2017; Quantin‐Nataf et al., 2021). However, the major issue present with this hypothesis is the large elevation distribution across LTR unit deposits. We observe an elevation change of 470 m throughout the study area (Table S1), a range not likely to occur from a localized flow deposit. Furthermore, the LTR unit drapes topographic variations. Lava flows typically infill topographic lows, whereas fall deposits (i.e., pyroclastic falls and ashfalls) drape over and conform to the underlying topography (Wright et al., 1980).

**Figure 7:**
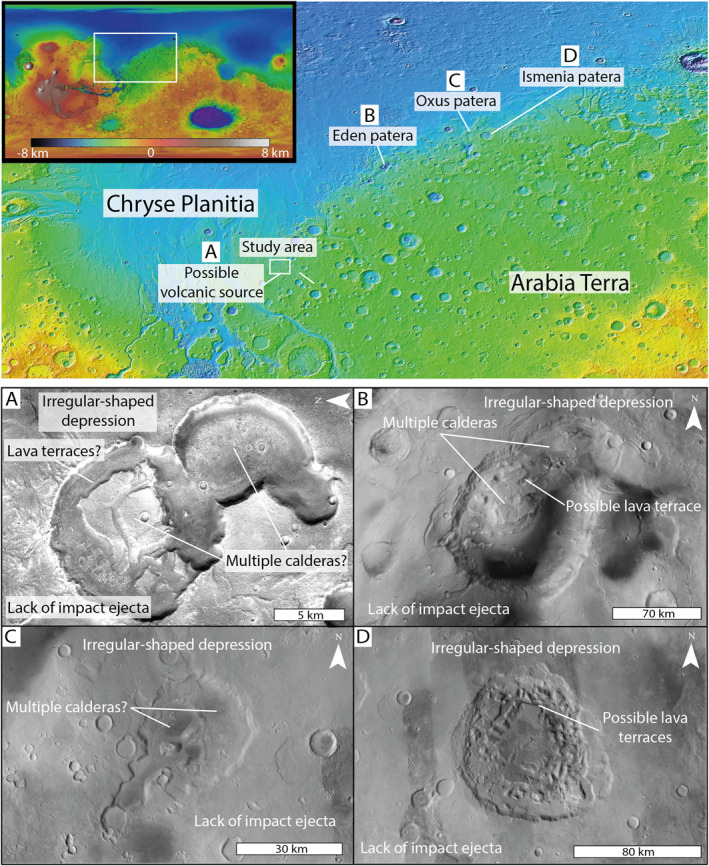
Possible sources of volcanic ash in western Arabia Terra. (a) A candidate‐ancient explosive volcano in Oxia Planum identified by morphological similarities to paterae craters as described by Michalski and Bleacher (2013) (CTX: N06_064538_1963). Images (b–d) are credited to: Global CTX Mosaic of Mars; NASA/JPL/MSSS/The Murray Lab. (b) Eden patera is the closest confirmed paterae crater to Oxia Planum and is a possible source of the LTR unit. (c) The irregular depression and lack of impact ejecta are suggestive of Oxus patera as an ancient explosive volcano. (d) Ismenia patera contains irregular features that have been interpreted as lava terraces (Michalski & Bleacher, 2013). We observe similar morphologies within the candidate‐explosive volcanic source in Oxia Planum.

An alternative interpretation is that the LTR unit is a volcanic ashfall deposit. One potential analog for the LTR unit is the circum‐Isidis capping unit near Jezero crater, which has a similar morphology and distribution (Figures 6g and 6h) and has been interpreted as a palaeo‐ashfall deposit (Hundal et al., 2022; Kremer et al., 2019). The circum‐Isidis capping unit is distributed over ∼70,000 km^2^ with a large topographic variation of the order of ∼4,000 m and a thickness of 2–23 m (Kremer et al., 2019). Ancient volcanic eruptions on Mars have been modeled with the potential to produce regional‐globally extensive airfall deposits (Brož et al., 2021; Kerber et al., 2011; Whelley et al., 2022). Modeling has previously suggested that distal pryoclasts from large‐scale volcanic provinces could have been dispersed over Arabia Terra (Kerber et al., 2012). However, a more local source of volcaniclastic material could be paterae that have been suggested to be remnant volcanoes. These features have been identified throughout Arabia Terra and could have produced highly explosive eruptions, resulting in unusually shaped paterae craters (Figure 7) (Chu et al., 2021, 2023; Michalski & Bleacher, 2013; Schaefer et al., 2022). Upon eruption, these may have formed 7,200 km^2^ of tephra (Michalski & Bleacher, 2013), with an estimated 1,000–2,000 caldera forming events occurring during the mid‐Noachian to early‐Hesperian (Whelley et al., 2022). Previously it has been suggested that the dispersal of pyroclasts from paterae could be a source of material for the equatorial layered deposits (ELDs) in Arabia Terra (Michalski & Bleacher, 2013; Whelley et al., 2021), with one study modeling the distribution of 12.5–25 m of volcanic ash across the Oxia Planum region from paterae eruptions (Whelley et al., 2021). Although it is worth noting that this is a contentious subject as other results suggest the layered deposits of Arabia were not formed by volcanic ash (Schmidt et al., 2021). However, these studies all focus on the distribution of layered, mantling deposits as opposed to dark, capping units such as the LTR. Eden patera (Figure 7b) is the closest of these possible volcanic features identified so far, at approximately 1,100 km from Oxia Planum, with modeling producing a range of volcanic ash dispersed over 7,000 km away from the source vent (Whelley et al., 2022). However, there is a crater within Oxia Planum that exhibits similarly unusual morphological traits—including lack of impact ejecta, nested craters, and lava terraces—to those of nearby paterae, including Eden patera, Oxus patera, and Ismenia patera (Figure 7). This potentially represents a more local volcaniclastic source within Oxia Planum and possibly for other sedimentary deposits in western Arabia Terra. The nature of the patera eruptions means they would be expected to have produced explosive volcanic eruption products (Chu et al., 2021, 2023; Michalski & Bleacher, 2013; Schaefer et al., 2022), and this local crater is therefore not a likely source of effusive volcanism, including lava.

Finally, we postulate that the LTR unit is an airfall deposit. This includes the aeolian redistribution of primary material including loess deposits and re‐worked volcanic ash. Possible loess deposits identified on Mars include the polar layered deposits (Tanaka, 2000), equatorial layered deposits (ELDs) in Arabia Terra (Annex et al., 2023), and “enigmatic deposits” that are more than 100 m thick and drape the topography. Authors speculate that these deposits may contain redistributed volcanic ash (Grant et al., 2010). We favor the airfall hypothesis as it can explain the relatively uniform thickness of the LTR unit, negating the effects of unit thickness with proximity to a possible local source of volcanic ash (e.g., Figure 7).

## 6 Toward an Interpretation on the Depositional Environment of the LTR Unit

Within this section, we aim to understand the regional palaeo‐environment of Oxia Planum at the time of the LTR unit formation. In previous sections, our analysis of the LTR unit, alongside comparison to terrestrial and martian analogs, suggests that the LTR unit is most likely an airfall deposit, possibly a primary or re‐worked volcanic ash. Possible sources include explosive volcanism associated with regional paterae craters in western Arabia Terra, or potentially a local source within Oxia Planum (Figure 7).

Such an airfall deposit would have blanketed the topography it was deposited on and has subsequently been preferentially preserved within palaeo‐topographic lows, where we observe the LTR unit as a capping unit atop inverted topography today. One major question is why the LTR unit is preserved with relatively high mechanical strength in select locations. Potential reasons for this selective preservation include localized protection from erosion, burial, preferential cementation in topographic lows—as discussed by Fawdon et al. (2022)—or a combination of these factors. Lithification of the LTR unit may have been aided by burial and could be an explanation for the polygonal fracturing seen throughout (Figure 3a). Previous work on the numerous mounds in and around Oxia Planum shows the LTR unit to drape across the mounds (McNeil et al., 2021), suggesting that the LTR unit was not significantly buried, and that our observations of polygonal fracturing here are likely a surface expression of the underlying phyllosilicate‐bearing unit. Alternatively, cementation could have acted as a precursor to lithification here. The process of cementation—the precipitation of minerals within pore spaces—removes the ability of a sediment to be transported away (e.g., through aeolian erosion), thus increasing sediment preservation (Day et al., 2019; Tian et al., 2018). On Earth, cementation typically occurs in areas of a high‐water table and where coarse‐sediment is abundant (Kocurek & Nielson, 1986; Simplicio & Basilici, 2015; Trewin, 1993). Identifying the source of the cementation is a key factor in further understanding the palaeo‐environment of Oxia Planum.

The LTR has previously been dated at 2.6 Ga using crater size‐frequency distribution (CSFD) (Quantin‐Nataf et al., 2021); however, this is possibly an unreliable method due to the small surface area of the LTR unit (Warner et al., 2015). Within this study, we were able to stratigraphically constrain the LTR unit between the phyllosilicate‐bearing unit and the sediment fan. The underlying phyllosilicate‐bearing unit has previously been dated at 4 Ga (Quantin‐Nataf et al., 2021) and the age of the sediment fan has previously been estimated at a minimum of >3.7–3.4 Ga based on CSFD of Kilkhampton crater ejecta that superposes the sediment fan (Gary‐Bicas & Rogers, 2021). Further study has concluded initial sediment fan formation in the mid‐Noachian based on catchment‐wide analyses (Fawdon et al., 2022). The stratigraphic relationship of these three units suggests an increase in the age of the LTR unit from the previously defined Amazonian aged (Quantin‐Nataf et al., 2021) to the late‐Noachian to early‐Hesperian. As previously discussed, the LTR unit likely occurred prior to deposition of, or during a hiatus in aqueous activity associated with, the sediment fan. Rather than being cemented through lake water‐sediment interaction, the cementation of the LTR unit can be explained by localized groundwater upwelling. Models of ancient groundwater on Mars predict a rising water table in the Noachian (Arvidson et al., 2006; Di Pietro et al., 2023) where topographic lows within Arabia Terra were likely to have contained water (Andrews‐Hanna et al., 2010). Groundwater may have infiltrated into topographic lows cementing the LTR unit preferentially here whilst in higher relief areas the LTR unit was subject to aeolian erosion and was not preserved.

The theory of groundwater cementation suggests that a mineral cement is the binding agent of the LTR unit. Previous work has detected mafic and phyllosilicate spectral signatures associated with LTR unit deposits in CRISM data, and interestingly, no hydrated minerals have been detected (Gary‐Bicas & Rogers, 2021; Quantin‐Nataf et al., 2021). The phyllosilicate spectral signatures could be associated with exposures of the underlying phyllosilicate‐bearing unit in areas of thinning LTR unit coverage (Figure 2d), or could be an expression of a phyllosilicate‐rich cement that has been found in the cementation of terrestrial volcaniclastics (Boggs, 2009). The mafic spectral signatures could be due to the volcanic origin of the LTR unit, but could also arise from mafic dust and sand trapping within the rugged and pitted surface. The lack of hydrated mineral detection could suggest concealment via dust coverage. Discrepancies between orbital and ground spectra have previously been identified along the traverse of the MSL *Curiosity* rover, where a strong ferric signature associated with hematite was identified from orbit, but on the ground was found to occur across a wider areal extent (Fraeman et al., 2020). Authors suggest this wider signature was not identified in CRISM data due to basaltic sand and dust coverage (Fraeman et al., 2020). Our observations suggest that the water‐table in Oxia Planum, and possibly the wider Arabia Terra region, was variable and fluctuating in the late‐Noachian and early‐Hesperian and was the cause of the preferential cementation of the LTR unit in topographic lows at Oxia Planum. These observations would be consistent with previous research that has summarized a fluctuating water table at Arabia Terra (e.g., Andrews‐Hanna et al., 2010; Salese et al., 2019; Schmidt et al., 2021) and the punctuated formation of the sediment fan at Oxia Planum (Fawdon et al., 2022).

## 7 Implications for the ExoMars Rover Mission

The ExoMars *Rosalind Franklin* rover has the mission goal of searching for signs of past or present life at Oxia Planum (Quantin‐Nataf et al., 2021; Vago et al., 2017), with the mission focusing on the collection of drill‐cores from the phyllosilicate‐bearing unit. The key to the fulfillment of this mission goal is the possible long‐term protection and preservation of remnant organic matter in the phyllosilicate‐bearing unit offered by the capping LTR unit. Here we have seen evidence of that protection, as most of the positive relief of the LTR unit can actually be attributed to the underlying phyllosilicate‐bearing unit. However, our study has shown that the LTR unit was likely only cemented in specific areas; therefore, a large majority of the phyllosilicate‐bearing unit is unlikely to have been protected by the LTR unit over time, as the uncemented sediment would have been easily eroded and removed by the wind. The locations with the greatest chance of fulfilling the ExoMars *Rosalind Franklin* mission goals are likely the phyllosilicate‐bearing strata exposed at the scarped edges of the LTR unit deposits that were protected over geologic time and may have more recently been exposed (Figure 5a).

Based on our assessment of the local stratigraphy, the LTR unit was likely deposited as an airfall—possibly a primary or reworked volcanic ashfall—during the late‐Noachian to the early‐Hesperian, during a hiatus in aqueous activity at Oxia Planum, which occurred after the deposition of the phyllosilicate‐bearing unit but before the formation of the sediment fan (Fawdon et al., 2022). The majority of Oxia Planum would have been arid during LTR unit deposition and a large, regional lake was probably not present during this time. The LTR unit may have been deposited into topographic lows that already contained a shallow layer of surface water due to groundwater infiltration, or a rising water table may have seen infiltrating groundwater shortly after deposition. This likely illustrates the episodic nature of the availability of water on early Mars (e.g., Kite, 2019; Kite et al., 2019; Simonetti et al., 2024). The exact nature of this depositional environment could be uncovered with the ExoMars *Rosalind Franklin* rover. Evidence of the LTR unit emplacement into topographic lows acting as shallow lacustrine environments could be identified with ripple marks, as has recently been explored by the MSL *Curiosity* rover at the marker band in Gale crater (Gupta et al., 2023). The ExoMars *Rosalind Franklin* rover will not be able to directly drive onto the LTR unit deposits due to its rugged terrain (Carter et al., 2016); however, remote sensing observations will be valuable to detect any bedding structures. The capacity of the LTR unit to form boulders means it is likely that we will observe eroded LTR unit rocks surrounding the deposits. These could be subject to further near‐field rover investigations including high resolution imaging and spectral analysis for comparison to orbital data analysis.

## 8 Conclusions

Our investigation of the LTR unit suggests that it most likely records an airfall deposit, possibly a palaeo‐ashfall, deposited onto the phyllosilicate‐bearing unit at Oxia Planum around the mid Noachian‐early Hesperian (∼4–3.7 Ga). The present‐day exposures of the LTR unit occur in discrete deposits interpreted as areas of ancient preferential cementation, likely due to groundwater infiltration into palaeo‐craters and other topographic lows, within an otherwise arid environment. In regions where LTR sediments were not cemented, they were likely not preserved in the geologic record. The sediment fan at the eastern margin of Oxia Planum overlies the LTR unit, suggesting that regional lacustrine processes prevailed at Oxia Planum after the LTR unit formation. As the phyllosilicate‐bearing unit is also associated with aqueous processes, the stratigraphic position of the LTR unit is consistent with the largely episodic nature of water availability on early Mars. Our findings suggest that the LTR unit may have contributed to the preservation of the underlying phyllosilicate‐bearing unit, but that this likely only occurred in areas of topographic lows where preferential cementation could occur. In these regions, up to decameter‐scale scarped cliffs of phyllosilicate‐bearing rock are exposed beneath the LTR unit. These exposures could be exploited to fulfill the mission goals of the ExoMars *Rosalind Franklin* rover at Oxia Planum.

## Conflict of Interest

The authors declare no conflicts of interest relevant to this study.

## Supporting information

Table S1

## Data Availability

The Mars orbital data products used here are available from the NASA Planetary Data System: (a) HiRISE (McEwen, 2007). (b) CTX (Malin, 2007). (c) MOLA (Smith et al., 2003). (d) THEMIS (Christensen, 2002). Other data products can be accessed through the ESA Planetary Science Archive in the following links: (a) HRSC (Neukum et al., 2004); https://pds‐geosciences.wustl.edu/missions/mars_express/hrsc.htm. (b) CaSSIS (Thomas et al., 2017). CTX and CaSSIS DTMs from Fawdon et al. (2021) can be found at 10.1080/17445647.2021.1982035. HiRISE DTMs from Tao et al. (2021) can be found at 10.3390/rs13214220. LANDSAT data were accessed through the United States Geological Society (USGS); https://www.usgs.gov/landsat‐missions/landsat‐data‐access. The data created for this project—including the shapefiles of the LTR unit at Oxia Planum, the MOLA point elevations of the LTR unit, the shapefiles of the location of the topographic profiles within Figure 5, and the raw data used to create these profiles—can be found within the Figshare archive (Harris, 2024).
