# Supplementary material for: A Low Albedo, Thin, Resistant Unit in Oxia Planum, Mars: Evidence for an Airfall Deposit and Late‐Stage Groundwater Activity at the ExoMars Rover Landing Site
Source: J Geophys Res Planets. 2024 Nov 22;129(11):e2024JE008527. doi: 10.1029/2024JE008527 (PMC11583114; doi:10.1029/2024JE008527)
Supplement: Supplementary file 1 — Table S1 [file JGRE-129-0-s001.docx]

**Supplementary Materials**

**Table S1** – Elevation of the LTR unit deposits in Oxia Planum using MOLA and HRSC point measurements (300 m/pixel).

| ID | Area (km^2) | Elevation (m) |
| --- | --- | --- |
| 1 | 4.27 | -2707 |
| 2 | 4.55 | -2805 |
| 3 | 21.82 | -2805 |
| 4 | 18.19 | -2981 |
| 5 | 19.93 | -2959 |
| 6 | 3.11 | -2825 |
| 7 | 4.76 | -2864 |
| 8 | 13.92 | -2942 |
| 9 | 4.02 | -2957 |
| 10 | 0.12 | -2962 |
| 11 | 0.77 | -3010 |
| 12 | 1.17 | -3038 |
| 13 | 0.09 | -2960 |
| 14 | 0.23 | -2973 |
| 15 | 0.66 | -2986 |
| 16 | 0.33 | -2994 |
| 17 | 0.74 | -3002 |
| 18 | 0.51 | -3004 |
| 19 | 0.6 | -3008 |
| 20 | 0.04 | -3001 |
| 21 | 1.91 | -3046 |
| 22 | 1.25 | -3046 |
| 23 | 14.56 | -3131 |
| 24 | 3.75 | -3054 |
| 25 | 50.03 | -3127 |
| 26 | 6.7 | -3101 |
| 27 | 1.02 | -3089 |
| 28 | 5.19 | -3097 |
| 29 | 2.2 | -2999 |
| 30 | 9.12 | -3012 |
| 31 | 9.93 | -3016 |
| 32 | 13.23 | -3016 |
| 33 | 85.93 | -3111 |
| 34 | 179.09 | -3097 |
| 35 | 16 | -3045 |
| 36 | 0.72 | -3012 |
| 37 | 1.57 | -3064 |
| 38 | 70.66 | -2970 |
| 39 | 147.52 | -2830 |
| 40 | 2.5 | -2686 |
| 41 | 16.7 | -3117 |
| 42 | 18.48 | -3108 |
| 43 | 236.68 | -3073 |
| 44 | 2.12 | -2904 |
| 45 | 10.84 | -2930 |
| 46 | 0.31 | -2852 |
| 47 | 4.08 | -2894 |
| 48 | 81.13 | -2931 |
| 49 | 32.89 | -2975 |
| 50 | 8.55 | -3032 |
| 51 | 0.55 | -3050 |
| 52 | 6.8 | -3041 |
| 53 | 63.13 | -2974 |
| 54 | 9.84 | -2930 |
| 55 | 8.43 | -2919 |
| 56 | 0.77 | -2954 |
| 57 | 9.8 | -2994 |
| 58 | 0.06 | -2951 |
| 59 | 1.12 | -2961 |
| 60 | 2.82 | -2962 |
| 61 | 0.7 | -2981 |
| 62 | 0.09 | -2977 |
| 63 | 0.13 | -2977 |
| 64 | 1.04 | -2987 |
| 65 | 0.65 | -2999 |
| 66 | 0.68 | -2925 |
